# Gut microbiota characteristics of colorectal cancer patients in Hubei, China, and differences with cohorts from other Chinese regions

**DOI:** 10.3389/fmicb.2024.1395514

**Published:** 2024-06-19

**Authors:** Jianguo Shi, Hexiao Shen, Hui Huang, Lifang Zhan, Wei Chen, Zhuohui Zhou, Yongling Lv, Kai Xiong, Zhiwei Jiang, Qiyi Chen, Lei Liu

**Affiliations:** ^1^Department of Gastrointestinal Surgery, Intestinal Microenvironment Treatment Center, The Central Hospital of Wuhan, Tongji Medical College, Huazhong University of Science and Technology, Wuhan, Hubei, China; ^2^School of Life Sciences and Health Engineering, Hubei University, Wuhan, China; ^3^Department of Colorectal Disease, Intestinal Microenvironment Treatment Center, Tenth People’s Hospital of Tongji University, Shanghai, China

**Keywords:** microbial carcinogenesis, colorectal cancer, microbiome, biomarkers, gut

## Abstract

The research on the correlation or causality between gut microbiota and the occurrence, development, and treatment of colorectal cancer (CRC) is receiving increasing emphasis. At the same time, the incidence and mortality of colorectal cancer vary among individuals and regions, as does the gut microbiota. In order to gain a better understanding of the characteristics of the gut microbiota in CRC patients and the differences between different regions, we initially compared the gut microbiota of 25 CRC patients and 26 healthy controls in the central region of China (Hubei Province) using 16S rRNA high-throughput sequencing technology. The results showed that Corynebacterium, *Enterococcus, Lactobacillus*, and *Escherichia-Shigella* were significantly enriched in CRC patients. In addition, we also compared the potential differences in functional pathways between the CRC group and the healthy control group using PICRUSt’s functional prediction analysis. We then analyzed and compared it with five cohort studies from various regions of China, including Central, East, and Northeast China. We found that geographical factors may affect the composition of intestinal microbiota in CRC patients. The composition of intestinal microbiota is crucial information that influences colorectal cancer screening, early detection, and the prediction of CRC treatment outcomes. This emphasizes the importance of conducting research on CRC-related gut microbiota in various regions of China.

## 1 Introduction

Colorectal cancer (CRC) is one of the most common types of digestive tract tumors. According to the GLOBOCAN2022 cancer database released by the International Agency for Research on Cancer (IARC), CRC currently ranks third in global incidence and second in mortality, accounting for 9.6% and 9.3% of the total cancer incidence and mortality, respectively. In China, the number of new cases and deaths from CRC in 2022 was 517,106 and 240,010, ranking first globally (accessed 2024.02.05)^1^. The incidence and mortality rates are higher than the global average and have been on the rise in recent years, with a trend toward affecting younger individuals (Siegel et al., 2019).

The incidence of CRC is not only related to uncontrollable factors such as age, personal medical history, and family history of hereditary gastrointestinal diseases, but also closely linked to controllable factors such as dietary patterns and lifestyle (Anwar et al., 2004; Sullivan et al., 2022). Unhealthy habits such as smoking, excessive alcohol consumption, and high consumption of red or unprocessed meat may increase the risk of developing CRC (Theodoratou et al., 2017; Keum and Giovannucci, 2019; Wong et al., 2019). Conversely, a diet abundant in fiber, polyunsaturated fatty acids, fruits, and vegetables, along with regular exercise, can reduce the risk of CRC (Baena and Salinas, 2015; Van Blarigan and Meyerhardt, 2015; Sánchez-Alcoholado et al., 2020). The occurrence of CRC may be the result of the combined effect of multiple risk factors, but its close relationship with lifestyle connects CRC to the gut microbiota (Nistal et al., 2015).

The development of metagenomic techniques has led researchers to focus on the gut microbiota. Extensive data from animal and human models has revealed a strong correlation between the gut microbiota and the onset of various gastrointestinal and extraintestinal diseases (Zhu et al., 2011; Losurdo et al., 2020; Miyauchi et al., 2023). The gut microbiota generally refers to the complex biological system within the gut, comprising various microorganisms such as bacteria, fungi, viruses, and protozoa (Falony et al., 2016; Cani, 2018). Under specific conditions, the gut microbiota maintains a relatively stable and dynamic equilibrium (Rinninella et al., 2019; Kurilshikov et al., 2021). However, unhealthy dietary habits and external factors such as diseases can lead to alterations in the composition and structure of the gut microbiota. This is primarily evident in the excessive growth of specific “harmful” microbial groups, such as polyketide synthase-positive *E. coli, enterotoxigenic Bacteroides fragilis, S. gallolyticus*, and *Fusobacterium nucleatum*, as well as the suppression of “beneficial” microbial groups, including *Clostridium butyricum, Streptococcus thermophilus, Lacticaseibacillus Paracasei, Ruminococcus gnavus*, and *Blautia producta*. This leads to an increase in the production of harmful metabolites, such as polyamines, and a decrease in metabolites that are beneficial for gut barrier protection, such as short-chain fatty acids (Weir et al., 2013; Louis et al., 2014; Sánchez-Alcoholado et al., 2020; Qu et al., 2023). This process thereby promotes the development of CRC. As mentioned by Tjalsma et al. (2012) alterations in the gut microbiota during the progression of CRC will promote the proliferation of “bacterial passengers,” opportunistic bacteria that contribute to cancer advancement. It is worth noting that *F. nucleatum*, which is commonly abundant in patients with CRC, has been identified as a “bacterial passenger,” and research has shown that it can serve as a prognostic biomarker (Yamaoka et al., 2018; Oh et al., 2019). Multiple studies have demonstrated that the gut microbiota not only plays an important role in the development of CRC but also can participate in regulating and improving the treatment process of CRC (Lang et al., 2023; Qu et al., 2023).

China is the most populous country in the world and also one of the countries with a high incidence of CRC (Shao et al., 2023). In China, CRC incidence and mortality vary widely among regions and provinces (Zhang et al., 2019; Xu et al., 2023). China’s diverse geographical environment, with its abundant habitats, influences unique eating habits that not only shape the gut microbiota with regional characteristics but also play a significant role in the varying incidence of CRC across different regions (Zhang et al., 2019; Lin et al., 2020; Yang et al., 2020; Wang et al., 2023; Xu et al., 2023). While there have been reports on the gut microbiota (GM) characteristics of CRC patients in various regions, there is currently no large-scale study focusing on the differences in GM among CRC patients in different regions of China (Jin et al., 2019; Yang et al., 2021; Yi et al., 2021; Li et al., 2022; Feng et al., 2023; Wang et al., 2023). Previous studies have primarily utilized sequencing techniques that target the V3-V4 region of the bacterial 16S rRNA. Therefore, this study initially analyzed the gut microbiota of CRC patients in the central region of China using high-throughput sequencing of the 16S V3-V4 region. Then, several representative external datasets from various regions of China were included for comparison to analyze the core microbiota of Chinese CRC patients and to examine the differences in gut microbiota among different regions.

## 2 Materials and methods

This study was conducted in accordance with the Helsinki Declaration and approved by the Ethics Committee of Wuhan Central Hospital (code: YLXL2023-046). All participants signed informed consent forms before taking part in the experiment.

### 2.1 Sample collection

From June 2023 to December 2023, 25 patients with CRC and 26 healthy controls were recruited from Wuhan Central Hospital (Supplementary Table 1). The CRC patients were from the department of gastrointestinal surgery and oncology and met the following inclusion criteria: (1) clinically diagnosed with CRC; (2) no family history of intestinal diseases; (3) aged 50–75 years; (4) had not received antibiotics or immunosuppressive therapy in the past 30 days; (5) had not undergone radiotherapy or chemotherapy before sampling. In addition, 26 healthy individuals of the same age as the CRC patients were included as controls to eliminate confounding factors such as lifestyle and diet. They met the following inclusion criteria: (1) no diagnosed diseases; (2) no use of antibiotics or probiotics in the past 30 days. The exclusion criteria for both groups were similar: (1) having diseases related to the intestinal microbiota, such as inflammatory bowel disease and other autoimmune diseases; (2) having a family history of intestinal diseases; and (3) being pregnant or lactating women. All fecal samples were collected from the patients before breakfast, immediately frozen in liquid nitrogen, and then transferred to −80°C for storage until DNA extraction.

### 2.2 DNA extraction and 16S rRNA gene sequencing

The fecal samples were collected and DNA was extracted using the HiPure Stool DNA Mini Kit (Magen, Guangzhou, China) according to the manufacturer’s instructions. PCR amplification was carried out using universal primers that target the V3-V4 hypervariable region of the 16S rRNA gene (341F: 5′-CCTACGGGNGGCWGCAG-3′ and 805R: 5′-GACTACHVGGGTATCTAATCC-3′). The PCR conditions were as follows: 95°C for 3 min, followed by 30 cycles of 95°C for 30 s, 55°C for 30 s, and 72°C for 30 s, with a final extension at 72°C for 5 min. The PCR products were purified using the AxyPrep DNA Gel Extraction Kit (Axygen Biosciences, Union City, CA, USA) after confirmation by 2% agarose gel electrophoresis. The quantification was performed using Qubit 4.0 (Thermo Fisher, USA). Sequencing was conducted on the Illumina MiSeq platform, producing 2 × 250 bp paired-end reads. The raw data has been submitted to the Genome Sequence Archive (GSA) at the China National Center for Bioinformation (CNCB) with the submission number CRA014969.

### 2.3 Bioinformatics and data analysis

The Qiime 2 platform was utilized to remove primers from the raw paired-end sequences in FASTQ format. Standard procedures are used, including filtering and trimming, denoising, and merging the reads into amplicon sequence variants (ASVs). The quality filtering of reads involves the ‘filterAndTrim’ function in DADA2, while the removal of chimeras utilizes the ‘removeBimeraDenovo’ function. The ASVs were subsequently mapped to the Silva reference database (version 138) for taxonomic annotation in order to analyze species differences among the samples (groups). The α-diversity indices, which include community richness, diversity, and evenness, were calculated using mothur. The assessment of β-diversity, which demonstrates differences in microbial community composition between different groups, was calculated using Principal Coordinate Analysis (PCoA) with the “pcoa” and “adonis” functions from the R package vegan. Statistical testing was performed using permutational multivariate analysis of variance (permanova). The default parameters of the PICRUSt2 software were utilized to predict metagenomes based on the ASV file and to investigate potential gene functions in the gut microbiota using the KEGG and COG database for comparison. The comparison of potential gene functions among groups was conducted using the Kruskal-Wallis test. Linear discriminant analysis effect size (LEfSe) was utilized to identify distinctive bacterial taxa (log LDA score >4 and *p* < 0.05) for comparison. R language (version 4.2.1) was used for visual data analysis. All values are presented as the mean ± standard deviation (SD). A significance level of *p* < 0.05 was deemed statistically significant.

### 2.4 Comparisons to external data

We searched the PubMed database for relevant studies on the fecal microbiome of Chinese colorectal cancer patients. All samples were collected prior to surgery or drug treatment, and we screened for raw data from high-throughput sequencing targeting the V3-V4 region of the 16S rRNA gene. We obtained the original sequencing data for these studies from the Sequence Read Archive (SRA) and Genome Sequence Archive (GSA). Data that were unavailable or ambiguously classified were not included in this study. In the end, a total of five studies were included. Among them, 3 cohorts were from the East China region (Shanghai, Shandong, and Jiangsu), 1 was from the Northeast region (Harbin), and 1 was from the Central China region (Anhui) (Jin et al., 2019; Yang et al., 2021; Yi et al., 2021; Li et al., 2022; Feng et al., 2023; Table 1). All sequences are processed and analyzed using the same workflow as in the previous step.

**TABLE 1:** Information on the research cohort included in this study.

References	DOI	Cohort	Group	CRC+HC (N)	Control (age and gender)	CRC (age and gender)	Disease information	TNM stage (I/II/III/IV)
Jin et al., 2019	10.1111/1462-2920.14498	Harbin	DBHEB	15+31	47 (23–64) (*n* = 31 21 male + 10 female)	63(54–83) (*n* = 15 10 male + 5 female)	Carcinoma patients (CRC)	Data not mentioned
Yang et al., 2021	10.1038/s41467-021-27112-y	Shandong	HDSD	54+146	60.46 ± 6.94 (*n* = 54, 26 male + 28 female)	62.42 ± 7.67 (*n* = 146 84 male + 62 female)	Sporadic CRC	33/50/57/6
Yi et al., 2021	10.1158/1078-0432.CCR-20-3445	Shanghai	HDSH	84+31	Data not mentioned	55.46 ± 9.47 (*n* = 84 58 male + 26 female)	Locally advanced rectal cancer	0/9/61/14
Li et al., 2022	10.3389/fcell.2022.916961	Wuxi	HDJS	82+0	No healthy controls	64.77 ± 0.92 (*n* = 98 58 male + 40 female) Only 82 stool samples included	Patients scheduled for colorectal resection (CRC)	10/51/30/7 (98)
Feng et al., 2023	10.3389/fmicb.2023.1034325	Anhui	HZAH	10+10	54.42 ± 11.40 (*n* = 10 5 male + 5 female)	57.05 ± 11.58 (*n* = 10 4 male + 6 female)	Colorectal cancer	0/6/4/0

## 3 Results

**FIGURE 1:**
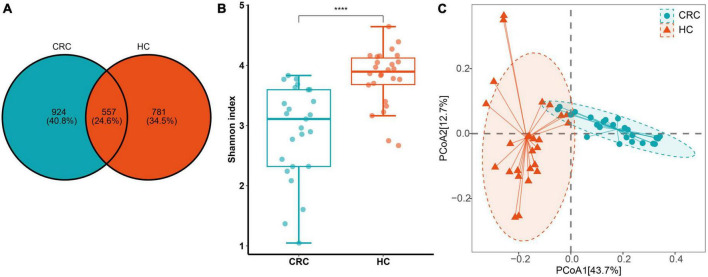
**(A)** ASV differences between CRC (Colorectal Cancer) and HC (Healthy Control) groups; **(B)** The Shannon index. **(C)** Principal coordinate analysis (PCoA) plots of gut microbiota in controls and CRC patients measured by the Bray-Curtis distance. ****p < 0.0001.

From a total of 51 fecal samples, 4,920,386 reads of the 16S rRNA gene were obtained. After quality filtering, 3,023,089 high-quality reads were obtained, averaging 59,276 reads per sample (Supplementary Table 2). Following the DADA2 quality control procedure, a total of 2262 ASVs were obtained for diversity and functional analysis, with 22 to 366 ASVs observed in individual samples. By comparing the ASV differences between the two groups using a Venn diagram, we found that the CRC group and the healthy control group shared 557 ASVs (24.8%) (Figure 1A). This indicates that CRC and healthy controls only share a small portion of gut microbiota.

### 3.1 Alpha and beta diversity of CRC and HC

In terms of α and β diversity between CRC and HC groups, the analysis of alpha diversity revealed that the CRC group exhibited a significantly lower Shannon index. This indicates a significant decrease in gut microbiota (GM) diversity in CRC patients compared to the healthy control group (Figure 1B). Using four algorithms (Bray-Curtis, abund-jaccard, unweighted-unifrac, weighted-unifrac) to analyze the β diversity differences between the CRC and HC groups, the results are displayed in Figure 1C, with Bray-Curtis used as an example. The PCoA clustering results revealed that samples from the CRC group and HC group clustered separately, exhibiting a distinct trend of spatial distance distribution. In addition, linear regression analysis based on the Shannon index showed that age had almost no impact on gut microbiota diversity in this study (Supplementary Figure 1A). In the gender grouping pattern, beta diversity results based on the Bray-Curtis distance algorithm and Anosim test indicated that gender also had no significant impact in this study (Supplementary Figures 1B, C).

### 3.2 Microbial profile of CRC and HC

**FIGURE 2:**
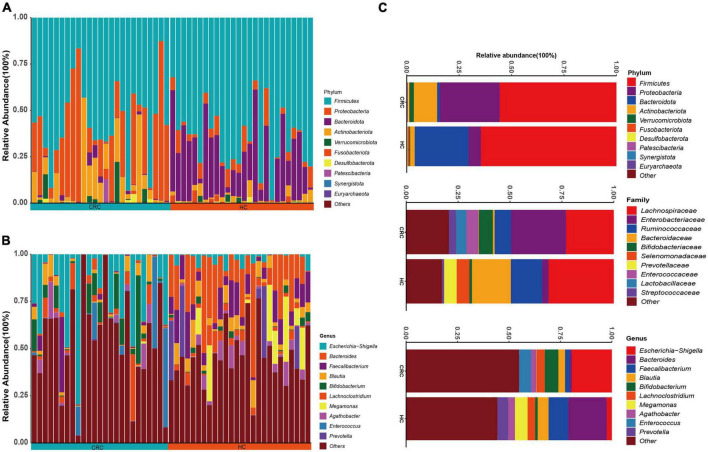
Relative abundance of intestinal bacterial communities at the phylum **(A)** and genus **(B)** levels in CRC patients and healthy control samples, as well as a comparison of the relative abundance of the two groups at the phylum, family, and genus levels **(C)**.

The species annotation results based on the SILVA database are depicted in Figure 2. A total of 13 phyla, 20 classes, 56 orders, 100 families, and 283 genera were identified in all samples. At the phylum level, both the CRC group and the HC group contained Actinobacteria, Bacteroidetes, Campylobacteria, Cyanobacteria, Desulfobacteria, Euryarchaeota, Firmicutes, Fusobacteria, Patescibacteria, Proteobacteria, Synergistetes, and Verrucomicrobiota. Among them, the phyla with an average relative abundance of more than 1% in the CRC group were Actinobacteria, Bacteroidetes, Firmicutes, and Proteobacteria, while in the HC group, the phyla with an average relative abundance of more than 1% were Actinobacteria, Bacteroidetes, Firmicutes, Proteobacteria, and Verrucomicrobiota. Firmicutes (>50%) had the highest proportion in both the CRC and HC groups. At the family level, the HC group had families with an average relative abundance of >5%, including Enterobacteriaceae, Lachnospiraceae, Ruminococcaceae, Bifidobacteriaceae, and Enterococcaceae, while the CRC group had families with an average relative abundance of >5%, including Lachnospiraceae, Bacteroidaceae, Ruminococcaceae, Selenomonadaceae, and Prevotellaceae. At the bacterial genus level, genera with an average relative abundance greater than 1% in both the CRC and HC groups included *Agathobacter, Anaerostipes, Bifidobacterium, Blautia, Escherichia-Shigella, Faecalibacterium, Lachnoclostridium, Roseburia, Ruminococcus, Streptococcus, Subdoligranulum, and [Ruminococcus]_torques_group.*

### 3.3 Differential bacteria in CRC compared to HC

**FIGURE 3:**
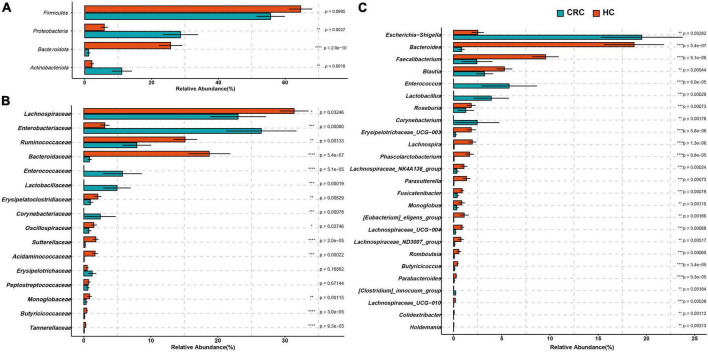
Comparison of differences in the abundance of most abundant bacteria at the levels of phylum **(A)**, family **(B)**, and genus **(C)** between the CRC and HC. *p < 0.05; **p < 0.01; ***p < 0.001; ****p < 0.0001.

At the phylum level, there was no significant difference in the abundance of Firmicutes between the two groups (FDR adjusted *p* > 0.05). However, Actinobacteriota, Desulfobacterota, and Proteobacteria were significantly more abundant in the CRC group, while Bacteroidota was significantly more abundant in the HC group (Figure 3A and Supplementary Table 3, all *p*-values were adjusted by FDR). At the family level, Corynebacteriaceae, Enterobacteriaceae, Enterococcaceae, and Lactobacillaceae were significantly more abundant in the CRC group, while Acidaminococcaceae, Bacteroidaceae, Butyricicoccaceae, Erysipelatoclostridiaceae, Marinifilaceae, Monoglobaceae, Ruminococcaceae, Sutterellaceae, and Tannerellaceae were significantly more abundant in the HC group (Figure 3B). Among these, Bacteroidaceae and Ruminococcaceae were significantly dominant families in the HC group, while Enterobacteriaceae and Enterococcaceae were significantly dominant families in the HCC group (>5%). Figure 3C displays 26 genera with significant differences between the CRC and HC groups after p-value correction. Among these, *Bilophila, Corynebacterium, Enterococcus, Escherichia-Shigella, Lactobacillus, and [Clostridium]_innocuum_group* were significantly enriched in the CRC group, while 20 genera, including *Bacteroides*, were significantly enriched in the HC group.

### 3.4 Analysis of classification biomarkers in CRC and HC using LEfSe

**FIGURE 4:**
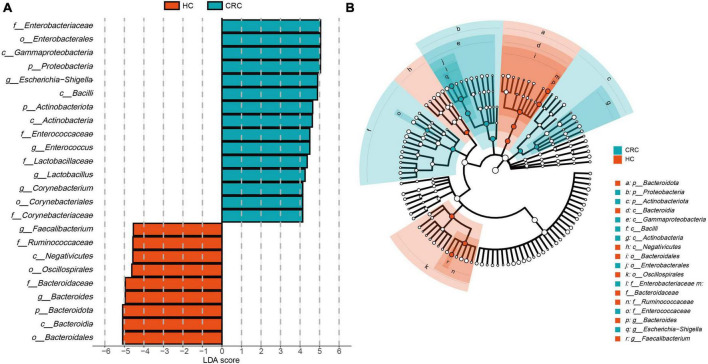
LEfSe analysis and LDA representation of microbiome features in CRC compared to healthy controls. **(A)** The histogram represents significantly different taxonomic units between CRC patients (blue) and healthy controls (HC) (red) based on effect size (LDA score [log 10] 4). **(B)** The cladogram shows differences in enriched taxa in CRC patients (blue) compared to enriched taxa in controls (red).

The estimated phylotypes of the microbial community in CRC patients and healthy controls were compared using LEfSe analysis. Based on LDA scores, the CRC group exhibited significantly higher relative abundance of Corynebacteriaceae, Enterococcaceae, Lactobacillaceae, Enterobacteriaceae, *Corynebacterium, Enterococcus, Lactobacillus*, and *Escherichia-Shigella* (LDA score [log 10] > 4). In contrast, the microbial community in healthy controls was characterized by Bacteroidaceae, Ruminococcaceae, *Bacteroides, Blautia*, and *Faecalibacterium* (LDA score [log 10] > 4) (Figure 4A). Overall, the CRC group has a distinct microbial community, characterized by differences from the HC group. These differences are primarily evident in the compositional variations of the major phylogenetic species in Bacteroidota, Firmicutes, Actinobacteriota, and Proteobacteria (Figure 4B and Supplementary Table 4).

### 3.5 Functional enrichment analysis and differences in pathway abundance between CRC patients and healthy controls

**FIGURE 5:**
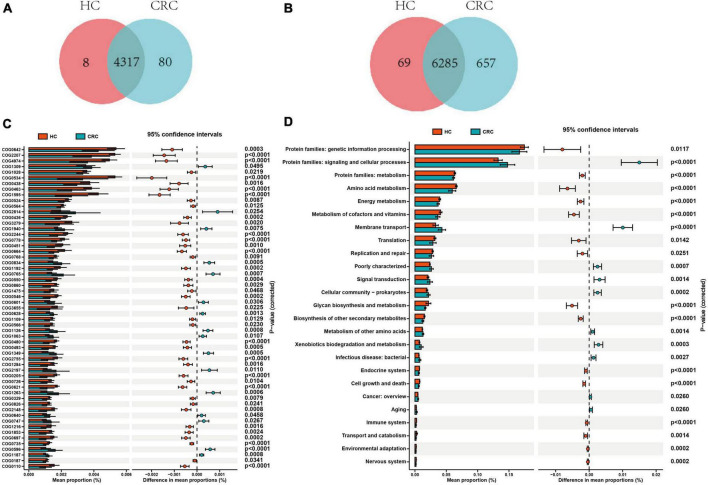
Comparison of functional prediction pathways between CRC and HC groups. Venn diagram illustrating the variances in the cluster of orthologous groups (COG) **(A)** and KEGG level 2 pathways **(B)** between CRC and HC groups. **(C)** The bar graph illustrates variations in the functional categories of COG in a vertical orientation. **(D)** The bar graph displays differences in the abundance of KEGG level 2 pathways. Differences between the two groups were compared using *t*-tests, with a *p*-value of <0.05 considered statistically significant.

Based on the 16S rRNA results of gut microbiota, functional prediction was performed using PICRUST2 software. The results of the Clusters of Orthologous Groups (COG) functional annotation showed that both CRC and healthy controls shared 4317 pathways, while CRC had 83 unique pathways. The Kyoto Encyclopedia of Genes and Genomes (KEGG) pathway annotation results revealed that both CRC and healthy controls shared 6285 pathways, while CRC had 657 specific pathways. Additionally, 8 pathways from COG and 69 pathways from KEGG were predicted to be present exclusively in the healthy controls (Figures 5A, B). Based on the functional annotation of COG differential genes, a total of 57 COGs showed significant differences between the CRC and HC groups, with 16 COGs significantly enriched in the CRC group (relative abundance > 0.1%, all *p*-values were adjusted by FDR, and *Q*-values were significant (i.e., *Q* < 0.05) (Supplementary Table 5). The KEGG pathway annotation results indicate that there are 25 significantly different metabolic pathways at the KEGG Level 2 between the CRC group and the HC group. These pathways include protein families: signaling and cellular processes, membrane transport, signal transduction, xenobiotics biodegradation and metabolism, cellular community - prokaryotes, poorly characterized, infectious disease: bacterial, metabolism of other amino acids, aging, and cancer: overview, which are significantly enriched in the CRC group (Figures 5C, D and Supplementary Table 6). The enrichment analysis of these pathways indicates that these differential functions may contribute to the variances between the CRC group and the HC group.

### 3.6 Network analysis reveals a unique micro-ecosystem in the CRC group

**FIGURE 6:**
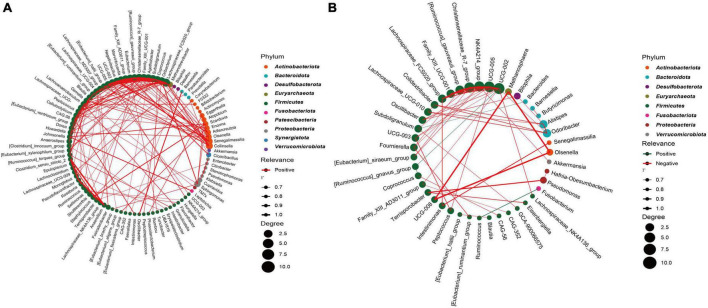
Highly correlated microbial networks at the genus level. Each circle represents a genus. Genera from the same phylum are marked with the same color. The size of the circle indicates the strength of the relationship with other species. Lines represent the correlation between two genera, with the thickness of the line representing the correlation coefficient. Red and green lines represent positive and negative correlations, respectively. **(A)** CRC group; **(B)** healthy control group.

To further analyze the bacterial interactions between the two groups, we constructed a network of bacterial interactions with relative abundance higher than 0.01% at the genus level using Spearman correlation analysis. Significant correlations (FDR adjusted *p* < 0.05; *r* > 0.6) were identified between 139 and 57 pairs of genera in the CRC and HC groups, respectively (Supplementary Table 7). In Figures 6A, B, we can observe that genera belonging to *Firmicutes* demonstrate relatively abundant correlation features in both groups, primarily displaying positive correlations. Specifically, in the CRC group, this is mainly manifested as interactions between *Firmicutes* and *Actinobacteriota*, as well as interactions within the *Firmicutes* genera. In the HC group, interactions mainly occur within the *Firmicutes* genera. The interaction relationship between *UCG-008, Methanosphaera*, and *Olsenella* is the most significant. In the CRC group, the top five genera in terms of correlation node count include *Lachnospiraceae_FCS020_group, Oribacterium, Subdoligranulum, Coprococcus*, and *Agathobacter*. In the HC group, the top five genera in terms of correlation node count include *UCG-002, UCG-005, NK4A214_group, Christensenellaceae_R-7_group*, and *[Ruminococcus]_gauvreauii_group*. It is worth mentioning that we found a significant negative correlation (*r* = −0.728) between *Ruminococcus* and *Fusobacterium* in the HC group, while no significant correlation with *Fusobacterium* was found in the CRC group. Although it was mentioned earlier that there was no significant difference in *Fusobacterium* between the two groups, Spearman’s correlation network analysis suggests a competitive inhibition relationship between *Ruminococcus* and *Fusobacterium* in the gut microbiota of the healthy control group.

### 3.7 Comparison with previous data

To compare the differences in gut microbiota among CRC cohorts from different regions in China, we initially compared the α-diversity of the present data with the previous dataset from various regions in China (Supplementary Figure 2A). We observed significant differences in α-diversity among the regional cohorts. The HZHB cohort exhibited the lowest Chao1 index compared to the other cohorts, while the HDSD cohort had the highest Chao1 index. It should be noted that the HDSD and HDSH cohorts have significantly larger sample sizes than the other cohorts. In addition, we found that the diversity of all samples in the various cohorts exhibited a consistent trend with the diversity of CRC samples in different regions.

**FIGURE 7:**
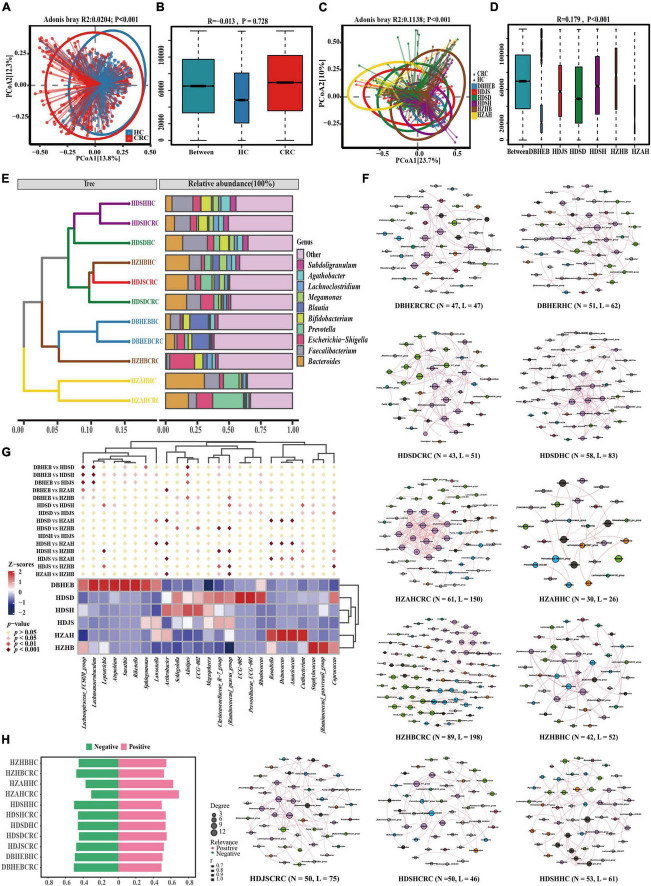
Gut microbiota of CRC patients and healthy controls in the present cohort and five other research cohorts. **(A)** PCoA plots of gut microbiota in all controls and CRC patients measured by the Bray-Curtis distance. **(B)** Between- and within-group differences in HC and CRC groups based on ANOSIM test. The boxplots from left to right represent the Bray-Curtis distance between HC and CRC samples, HC samples, and CRC samples, respectively. *R*- and *p*-values show community changes between compared groups. **(C,D)** The PCoA clustering results among the six regional cohorts and the ANOSIM test results among all groups use the same algorithm as before. **(E)** UPGMA clustering tree was constructed based on the top 10 genera with the highest average relative abundance, utilizing the Bray-Curtis distance algorithm. **(F)** Co-occurrence network diagram of species at the genus level in CRC and HC groups (Spearman *r* > 0.6 and FDR adjust *p* < 0.05). Nodes are colored according to their modularity attributes. Node size indicates the number of associative relationships with other species. Lines in the graph represent the correlation between two genera, and the thickness of the line corresponds to the correlation coefficient. Red and green lines represent positive and negative correlations, respectively. **(G)** A cluster heatmap constructed based on combinations of the top five genera with the highest number of nodes screened in different CRC cohorts. Diamond markers indicate the results of pairwise comparisons, with significance distinguished by color. **(H)** Display the proportion of positive and negative correlations of genus in all cohorts without conducting *p*-value correction and correlation coefficient filtering.

Next, for the principal coordinates analysis of these various datasets, we utilized the Bray-Curtis distance algorithm. First, we divided all samples into two groups based on CRC and HC after mixing them together. Although the analysis based on Adonis showed a *p*-value of <0.001 (Figure 7A) between the two groups, we noticed that this difference was caused by deviations in the clustering centers. Supplementary ANOSIM test results confirmed that the differences within groups, based solely on disease grouping patterns, were greater than those between groups (Figure 7B). So we regrouped according to different regions. Since the sample distribution of different regional cohorts is mainly concentrated in the East China region, the clustering centers will tend to be biased toward the East China samples. Although this may introduce some degree of bias in the analysis process, we can still observe the clustering trends among cohorts from different regions (Figure 7C). For example, there is a separation trend between the HZAH and HZHB cohorts in the central China region, while the HDSH and DBHEB cohorts show a more similar clustering pattern. The analysis results, based on Anosim, indicate significant differences (*P* < 0.001) in the spatial structure of gut microbiota among samples from different regions (Figure 7D). Further pairwise comparisons reveal that, apart from the lack of significant differences between HZAH vs HZHB (*R* = 0.07, *P* = 0.111), HZAH vs HDJS (*R* = −0.032, *P* = 0.692), and HZAH vs HDSD (*R* = 0.001, *P* = 0.477), all other inter-group comparisons indicate significant differences among samples from different regions (Supplementary Figure 3 and Supplementary Table 8). These results suggest that the differences in gut microbiota structure based on geographical location may play a more dominant role than the differences between CRC and healthy populations.

To further elucidate the variation in gut microbiota structure associated with geographic location and cancer, we analyzed the composition of the gut microbiota in various cohorts. The results of the genus-level Venn diagram revealed that the various groups had their own distinct genera (Supplementary Figure 2B). The HDSD cohort and the HZAH cohort had the highest numbers, with 459 and 113 specific genera, respectively. Only 116 genera were common to all groups, representing 20.17–38.28% of all genera in each group. This indicates that there are geographical variations in the composition of the gut microbiota at the genus level among the different regional cohorts.

To analyze this differential factor at the species’ evolutionary level. We constructed a UPGMA clustering tree (top 10 genera) using the Unweighted Unifrac distance algorithm (Figure 7E). The results of facts and speculations are similar. We did not observe the phenomenon of CRC and HC clustering separately, but it seems to be more influenced by geographical factors. We can clearly see that in the HDSH, DBHEB, and HZAH cohorts, CRC and HC groups cluster together, although not all. This clustering result indicates that the composition structure of the intestinal microbiota is influenced not only by diseases but also by geographical factors.

Genus-level co-occurrence network analysis reveals distinct connectivity patterns in various communities (Figure 7F). In the network correlation without filtering by *p*-values and correlation coefficients, we can observe that the number of positive correlations is slightly higher than negative correlations (Figure 7H). This suggests a relatively significant synergistic interaction among the main bacterial communities and a smaller antagonistic competitive effect. The most significant synergistic effect is found in the gut microbiota network of HZAHCRC (69.10%). After filtering and correcting for *p*-values and correlation coefficients, the threshold filter removed species that were weakly correlated, with a correlation coefficient higher than 0.6. We can see that almost all edges in all groups exhibit positive correlations, with only a few showing negative correlations. Among them, HZHBCRC has the most nodes (*N* = 89) and edges (*N* = 198), showing a rich interaction network; HZAHCRC has a high average degree (4.918), average weighted degree (4.795), graph density (0.082), and average clustering coefficient (0.8), indicating that the interactions between genera in this group are more frequent and close (Supplementary Table 9). In order to further explore this difference, we selected the top five ranked genera in all CRC cohort networks, which represent the most core genera in each group. The clustering heatmap results show that the three cohorts in the East China region, the two cohorts in the Central China region, and the DBHEB cohort are clustered into separate clusters (Figure 7G). Specifically, there are significant differences in the representative genera across CRC cohorts in different regions. Among them, *Alistipes, UCG-002, Christensenellaceae_R-7_group*, and *[Ruminococcus]_gnavus_group* are among the top 30 genera in average relative abundance in all CRC cohorts. These genera are predominantly enriched in the three cohorts in the East China region. The results above indicate that cohorts in different regions exhibit distinct species clustering network patterns. Particularly, the clustering outcomes in CRC cohorts suggest that the composition of intestinal microbiota in CRC patients may be influenced by geographical factors.

## 4 Discussion

The gut microbiota plays a crucial role in the onset and progression of CRC (Chen and Li, 2020; Taghinezhad et al., 2021; Ghorbani et al., 2022; Koyande et al., 2022; Feizi et al., 2023; Thoda and Touraki, 2023; Zheng et al., 2023). The gut microbiota, which consists of a diverse range of microorganisms including bacteria, fungi, viruses, and protozoa, not only plays a role in digesting and absorbing nutrients but also produces metabolites that could impact the development of intestinal cancer through various mechanisms (Ferranti et al., 2014; Gao et al., 2017; Meng et al., 2018). For instance, certain bacteria in the gut microbiota can generate metabolites such as lipopolysaccharide and extracellular enzymes. These substances can trigger abnormal proliferation and inhibit apoptosis of intestinal epithelial cells, potentially leading to mutations and the cancerous transformation of these cells (Zhu et al., 2013; Hur and Lee, 2015). Additionally, the gut microbiota can impact the onset of intestinal cancer by modulating the host’s immune response balance. Some studies have shown that specific bacterial strains can activate the host’s immune system and enhance the anti-tumor immune response, thus inhibiting the development and advancement of bowel cancer (Chen and Li, 2020; Taghinezhad et al., 2021; Ghorbani et al., 2022; Thoda and Touraki, 2023).

An imbalance in the intestinal microecology is closely associated with the development of CRC. Our findings suggest that the diversity of the gut microbiota is significantly lower in CRC patients compared to healthy controls. While a small number of studies did not find significant differences in diversity between patients with advanced precancerous lesions and healthy controls, the majority of current research supports the idea that gut microbiota diversity is reduced in CRC patients (Ahn et al., 2013; Feng et al., 2015; Yachida et al., 2019; He et al., 2021; Torres-Maravilla et al., 2021). However, Liu et al. (2023) found a greater diversity of fecal microbial communities in patients with stage III-IV CRC compared to patients with stage I-II CRC. This variation in species diversity may be attributed to a range of factors, including sample size, dietary patterns, ethnicity, and geographic location (Bultman, 2017; Tortora et al., 2022; Cai et al., 2023).

The results of three different levels of analysis based on phylum, family, and genus showed differences in the distribution of gut microbial composition between the CRC and HC groups. The results of Lefse-based differential analysis indicated that at the genus level, *Escherichia-Shigella, Corynebacterium, Enterococcus*, and *Lactobacillus* characterized the differential microbial composition in the CRC group. The involvement of *Escherichia-Shigella* and its metabolites in the mechanisms of intestinal inflammation development has been recognized as potential pathogens of CRC in past studies. They are also identified as key biomarkers for the diagnosis of CRC in the study by Liu et al. (2019), Fan et al. (2021), and Lu et al. (2023). Currently, there are not many reports related to *Corynebacterium* in CRC. In a recent study, the genus was found to be more prevalent in invasive CRC compared to patients with typical CRC (Zorron Cheng Tao Pu et al., 2020). In the human intestinal environment, *E. faecalis* is one of the most common species of *Enterococcus.* The role in CRC appears to be currently controversial (Gu et al., 2023). Although some studies have shown that *E. faecalis* is detected at high levels in patients with CRC and can contribute to the development of CRC through the production of reactive oxygen species (ROS), there have also been studies suggesting its potential anti-inflammatory effects and its use as a probiotic in the treatment of a variety of diseases (Huycke et al., 2002; Balamurugan et al., 2008; Zhou et al., 2016; Gong et al., 2017; Gu et al., 2023). *Lactobacillus*, as a major representative of probiotic bacteria, is known for its various anti-inflammatory and anti-tumor activities (Chen et al., 2022; Ghorbani et al., 2022). However, in our study, this genus exhibited an abnormal increase in the CRC group, which is consistent with the findings of a recent controlled study by Hu et al. (2022). This contradiction with the results of other studies may be related to sample size as well as environmental factors. The main differentially dominant genera in the HC group included *Blautia, Faecalibacterium*, and *Bacteroides*. *Blautia* plays a potential probiotic role by producing high concentrations of acetic acid *in vivo*. This strengthens intestinal epithelial cell tight junctions, blocks pathogenic bacterial infections, and has an improved prognostic impact on colon cancer (Fukuda et al., 2011; Chen et al., 2012). *Faecalibacterium* has shown positive effects in a variety of diseases such as inflammatory bowel disease (IBD), CRC, and Alzheimer’s disease (AD) (Martín et al., 2023). Among them, *Faecalibacterium prausnitzii* is an important bacterium in the human gut that produces butyric acid, protecting the digestive system from intestinal pathogens (Miquel et al., 2015; Zhou et al., 2018, 2021). An increased abundance of Bacteroides may trigger inflammatory effects and contribute to the development of diseases such as insulin resistance and obesity (Grasset et al., 2017; De Faria Ghetti et al., 2018; Lieber et al., 2019). Among them, *Bacteroides fragilis*, which is thought to have anti-inflammatory effects, is the more widely reported species. However, it is important to note that its subspecies can produce *B. fragilis* toxins that promote the development of colon tumors (Matsumiya et al., 2023). The results of different cohorts often vary due to sample size, sequencing technology, and geographic or racial factors. Further in-depth investigation is necessary to determine how to obtain key microbial markers of CRC with sufficient applicability.

Differential functional analysis revealed that, despite the CRC group having a greater number of unique microbiota, gene enrichment results suggested a decreasing trend in most pathways associated with CRC. Only a few pathways, such as signaling and cellular processes, membrane transport, signal transduction, and xenobiotics biodegradation and metabolism, were relatively more enriched in the CRC group. Such changes in metabolic pathways, which may be associated with altered microbial communities, can directly affect cancer cell metabolism. For example, aberrant activation of the PI3K/AKT/mTOR pathway promotes abnormal metabolism in cancer cells (Polivka and Janku, 2014; Wang et al., 2014). Overexpression of glucose transporter proteins (GLUTs) can increase glucose uptake, providing an additional source of energy and carbon to meet the rapid proliferation needs of cancer cells (Macheda et al., 2005; Yang et al., 2017). Abnormal expression of NF-κB and STAT3, which are closely related to inflammation and metabolic abnormalities in cancer cells, can affect crucial processes such as glucose metabolism, lipid metabolism, and amino acid metabolism (Fan et al., 2013; Vaiopoulos, 2013; Liu et al., 2014). Xenobiotics biodegradation and metabolism-related pathways play a crucial role in preventing or minimizing the harmful effects caused by external substances (Jin et al., 2023). During metabolism in cancer cells, certain enzymes (e.g., the cytochrome P450 (CYP) enzyme family) can transform drug and toxin molecules into compounds that are more readily excreted and eliminated (Go et al., 2015; Singh et al., 2023). In addition, it has been suggested that bacteria belonging to the phylum Aspergillus and Actinobacteria may metabolize terpenoids in tumor-adjacent tissues through biodegradation (Sun et al., 2023). However, further exploration in future studies is needed to understand the interaction of these pathways and their impact on the metabolism of cancer cells by regulating metabolism-related pathways.

Species correlation analysis networks suggest that the CRC group has relatively complex microbial role relationships. Although 16S rRNA-based results only allow precise identification of microorganisms down to the genus level, *Fusobacterium nucleatum*, an important member of the *Fusobacterium* genus, deserves our attention as a significant risk indicator for CRC. *F. nucleatum* acts as an opportunistic pathogen, invading the gut from the oral cavity through the gastrointestinal tract. It opportunistically invades the intestinal tract through the digestive system, starting from the mouth. *F. nucleatum* selectively colonizes CRC primarily through the bloodstream. It plays a role in colorectal carcinogenesis, metastasis, drug resistance, and immune microenvironment regulation through various complex biological mechanisms (Bullman et al., 2017; Abed et al., 2020; Meng et al., 2021; Wang and Fang, 2023). The results of this study suggest that the *Ruminococcus* in the HC group may exhibit competitive inhibitory effects on *Fusobacterium*. *Ruminococcus* is a significant member of the *Lachnospiraceae.* The bacterial species in this family not only possess the ability to reduce inflammatory markers but also can enhance the tumor immune surveillance activity of CD8^+^T cells and prevent carcinogenesis (Simpson et al., 2022; Taglialegna, 2023). The latest research results by Zhang et al. (2023) confirmed that *Ruminococcus gnavus* can inhibit tumor growth associated with *F. nucleatum*.

Comparative results from cohorts in various regions of China indicate that regional factors significantly influence the composition of intestinal microbiota in colorectal cancer patients. A recent review by Wang et al. (2023) summarized studies on colorectal cancer and the gut microbiota in China. The review revealed that Chinese patients with colorectal cancer have a more distinctive gut microbiota profile compared to those in other countries. This distinction is strongly associated with ethnicity and geographic location. Among the several study cohorts we included, colorectal cancer (CRC) patients in the HDSD cohort exhibited relatively high microbial diversity. In contrast, CRC patients in the HZHB region exhibited lower gut microbial diversity compared to other regions, which may be closely associated with the dietary habits prevalent in these regions (Zhang et al., 2015; Ren et al., 2023). In particular, the highly clustered results of the CRC group and HC group in the DBHEB and HDSH cohorts, respectively, may be related to the dietary patterns rich in red meat in northern China and the sweet dietary habits in Shanghai. However, we also noticed that there are no significant differences between certain regions, such as the HZAH cohort and the HZHB, HDJS, and HDSD cohorts. This lack of variation may be attributed to the geographical proximity of the province to these three regions. It should be pointed out that due to the lack of specific information such as age and gender of corresponding samples in other datasets, we only listed this information based on references, but cannot conduct statistical difference comparisons. Therefore, although our study seems to support the hypothesis that the gut microbiota of CRC patients is influenced by geographical factors, we still lack more detailed clinical sample information to substantiate this claim.

Although this study is an appropriate expansion of the research on intestinal flora in Chinese CRC patients, it still has many limitations and shortcomings. These include: (1) bias in sample size and sample distribution; (2) insufficient regional coverage; (3) difficulty in removing the batch effect of different cohort studies; (4) the effects of age, gender, and diet have not been evaluated; and (5) the limitations of 16S rRNA sequencing technology for species and even strain level identification. These factors should be taken into account in future studies. Large-scale multi-center studies should be conducted, utilizing macro-genomic sequencing and metabolomics technologies to thoroughly analyze the differences in species composition and metabolites of the intestinal microbial composition of CRC patients. This will offer scientific guidance to advance the utilization of intestinal microbiota in colorectal cancer screening, prognosis, and predictive biomarkers.

In this study, we analyzed the gut microbial diversity, species composition, and potential functions of CRC patients and healthy controls in Hubei, China, based on 16S rRNA gene sequencing data. We found that CRC patients had significantly higher levels of *Corynebacterium*, *Enterococcus, Lactobacillus*, and *Escherichia-Shigella* compared to the healthy population. Further multi-cohort studies indicate that the gut microbiota of CRC patients in different regions of China show geographical variations. In some regions, geographical factors may have a more significant impact on the gut microbiota than CRC.

## Data availability statement

The datasets presented in this study can be found in online repositories. The names of the repository/repositories and accession number(s) can be found in this article/Supplementary material.

## Ethics statement

The studies involving humans were approved by the Ethics Committee of Wuhan Central Hospital. The studies were conducted in accordance with the local legislation and institutional requirements. The participants provided their written informed consent to participate in this study.

## Author contributions

JS: Data curation, Funding acquisition, Investigation, Project administration, Resources, Supervision, Writing – original draft, Conceptualization. HS: Conceptualization, Data curation, Formal analysis, Investigation, Methodology, Project administration, Supervision, Writing – original draft. HH: Data curation, Formal analysis, Investigation, Methodology, Validation, Writing – original draft. LZ: Data curation, Formal analysis, Investigation, Methodology, Software, Validation, Writing – original draft. WC: Data curation, Formal analysis, Investigation, Methodology, Software, Validation, Writing – original draft. ZZ: Data curation, Formal analysis, Investigation, Methodology, Software, Validation, Writing – original draft. YL: Data curation, Formal analysis, Investigation, Methodology, Project administration, Software, Writing – original draft. KX: Data curation, Investigation, Methodology, Software, Validation, Visualization, Writing – original draft. ZJ: Data curation, Investigation, Methodology, Software, Validation, Visualization, Writing – original draft. QC: Conceptualization, Data curation, Formal analysis, Methodology, Project administration, Supervision, Validation, Writing – review and editing. LL: Conceptualization, Data curation, Formal analysis, Funding acquisition, Methodology, Project administration, Resources, Supervision, Writing – review and editing.

## Footnotes

1
https://gco.iarc.fr/
