# Supplementary material for: Gut microbiota characteristics of colorectal cancer patients in Hubei, China, and differences with cohorts from other Chinese regions
Source: Front Microbiol. 2024 Jun 19;15:1395514. doi: 10.3389/fmicb.2024.1395514 (PMC11220721; doi:10.3389/fmicb.2024.1395514)
Supplement: Supplementary file 1 [file Data_Sheet_1.docx]

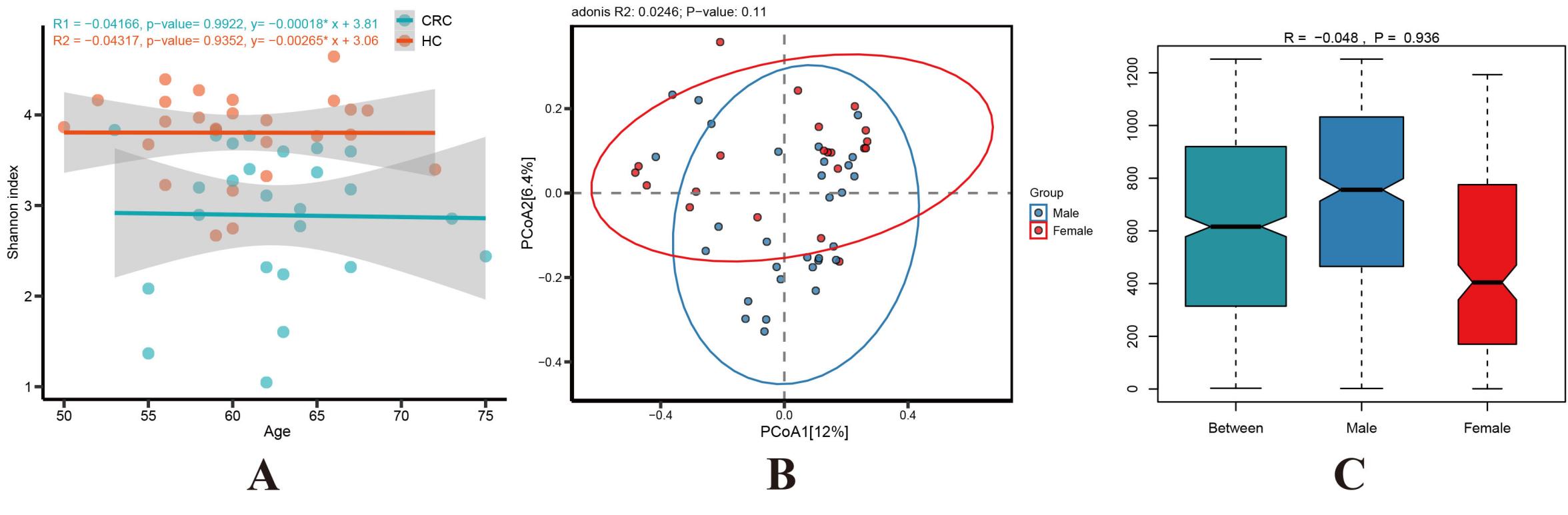
Figure S1. A) Age-related shannon index linear regression analysis in CRC (Colorectal Cancer) and HC (Healthy Control) groups; B) PCoA clustering results based on gender grouping (Bray-Curtis); C) Between- and within -group differences in Male and Female based on ANOSIM test.
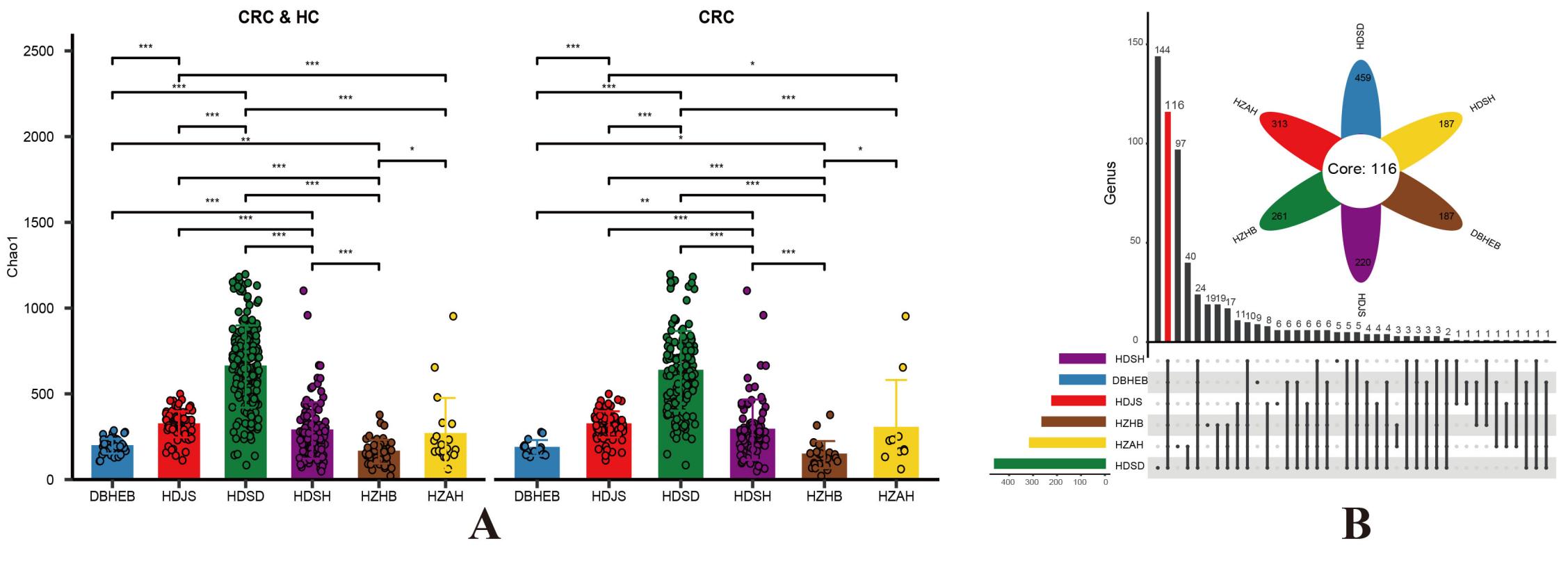


Figure S2. Gut microbiota of CRC patients and healthy controls in the present cohort and five other research cohorts. (A) Chao1 index of all samples and only in CRC patients. (B) Venn and upset diagrams illustrate shared genera among different cohorts and the overlap between cohorts. *, *p* < 0.05; **, *p* < 0.01; ***, *p* < 0.001.


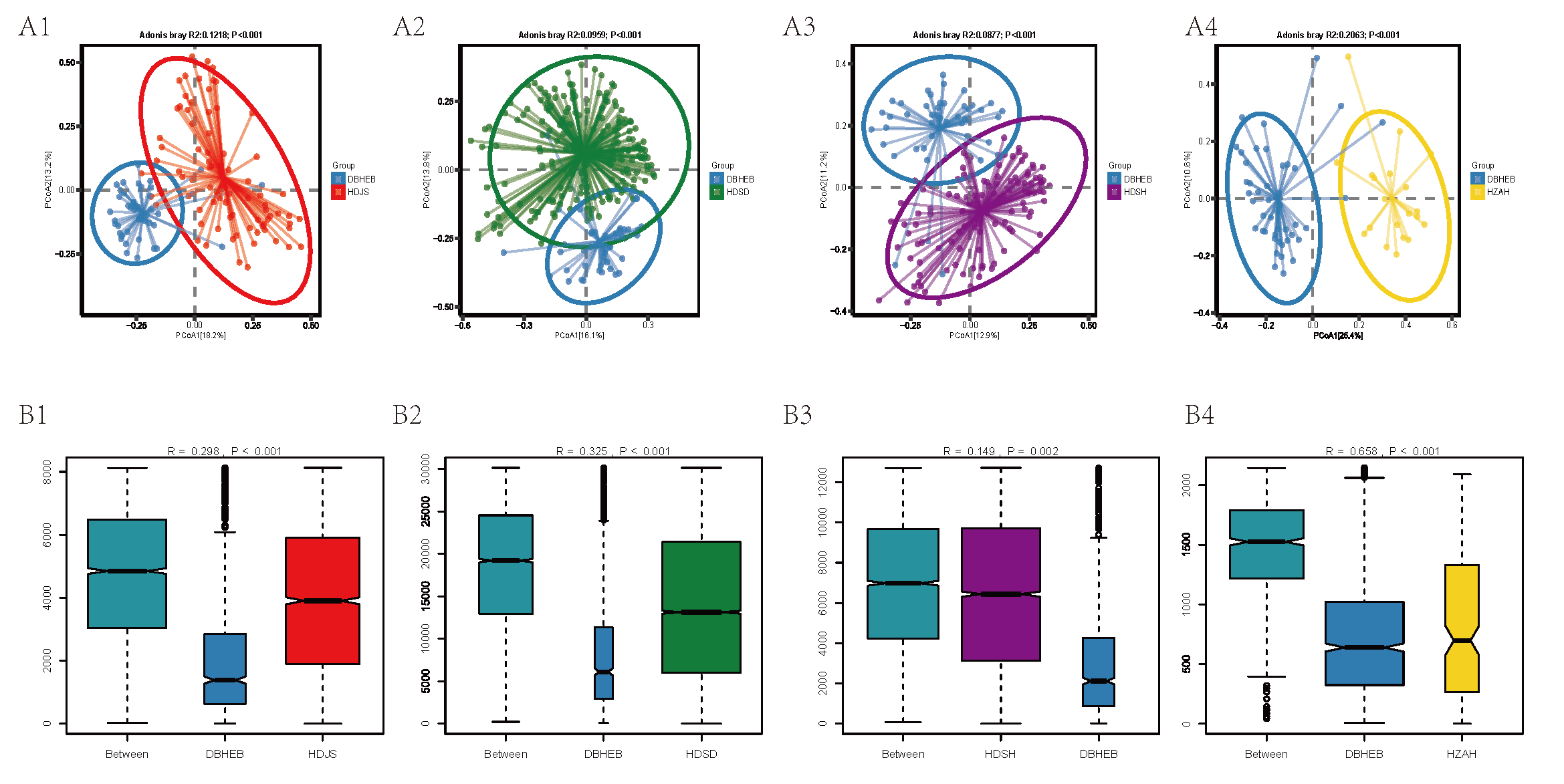


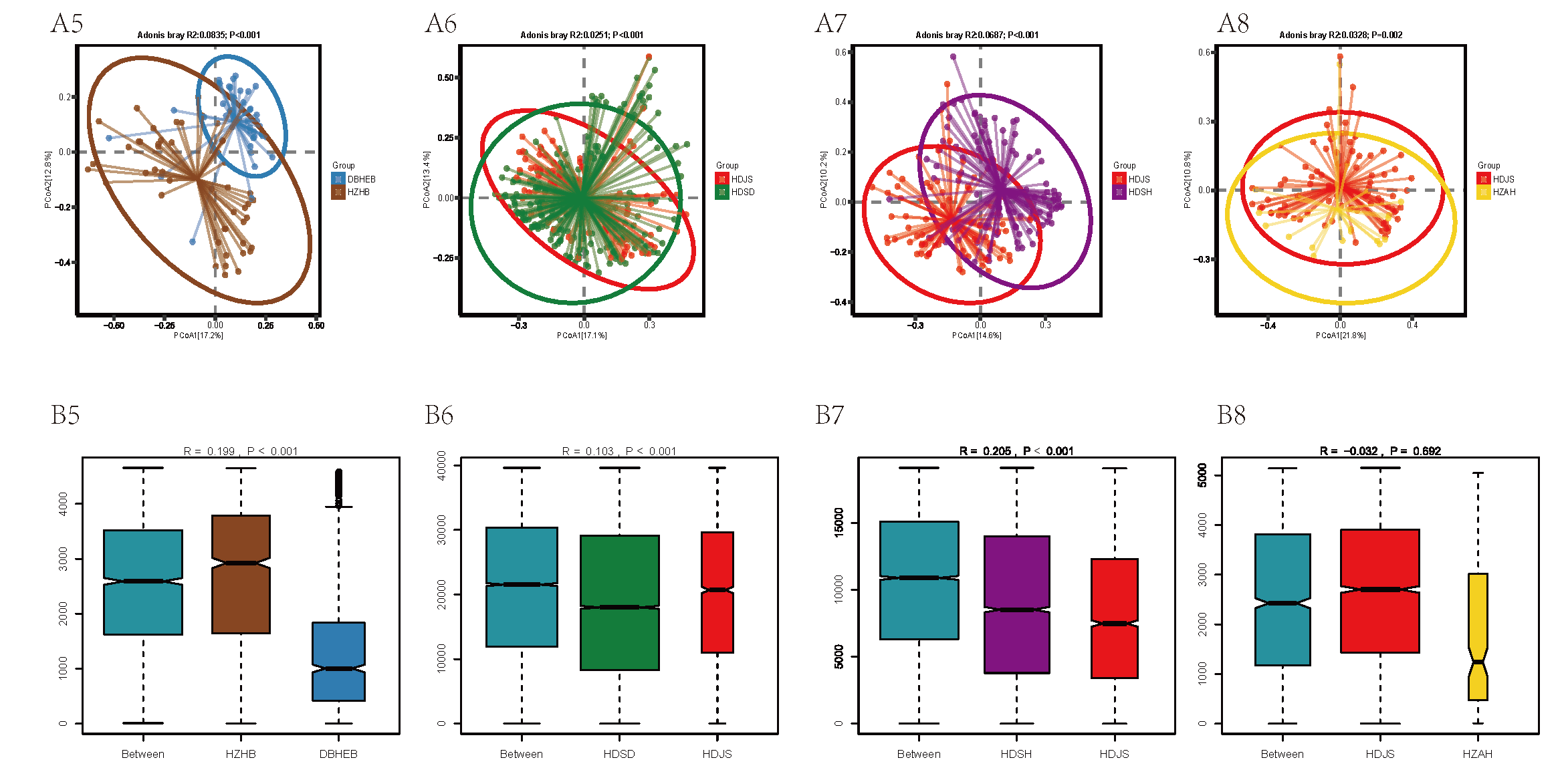

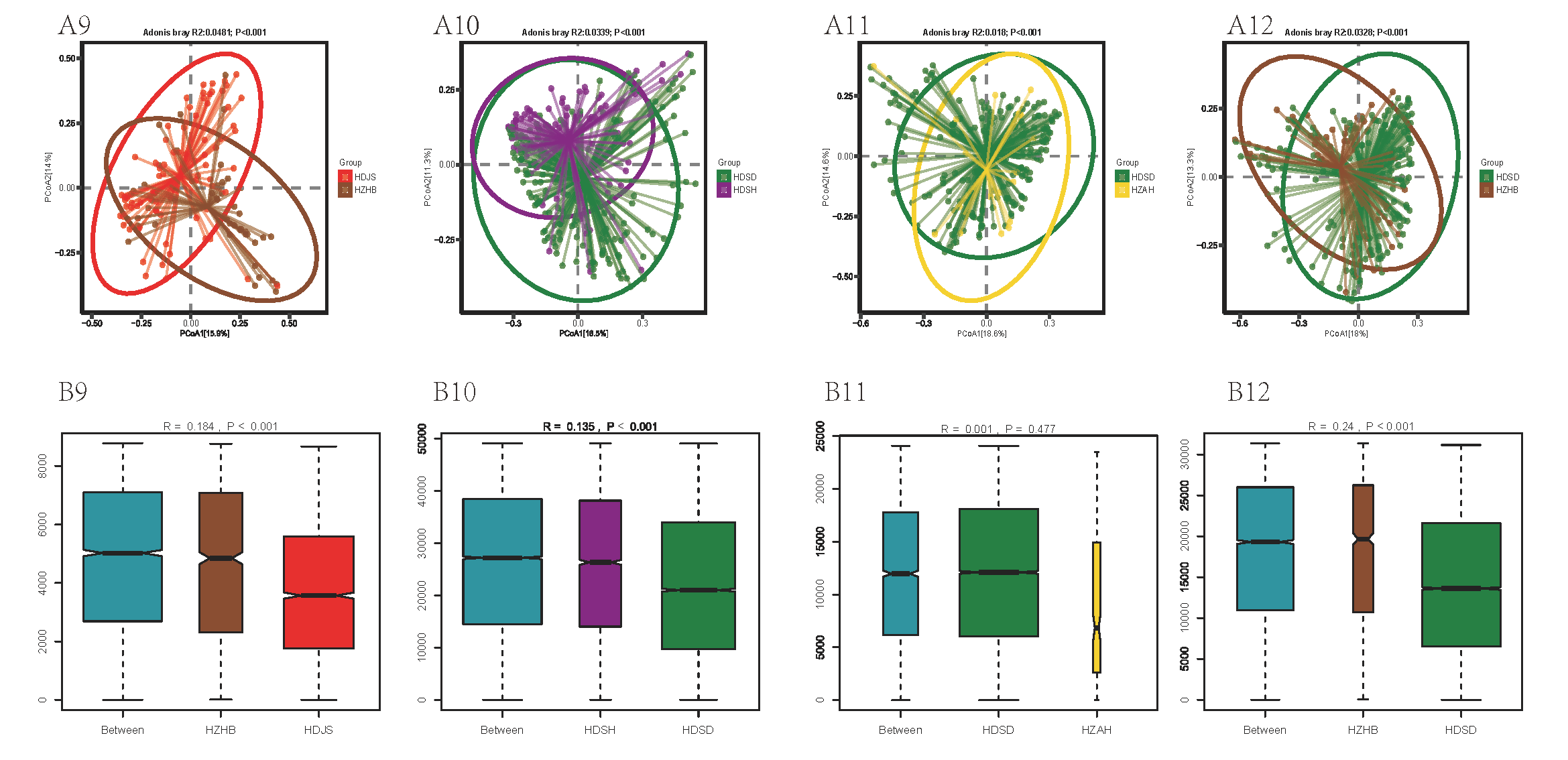

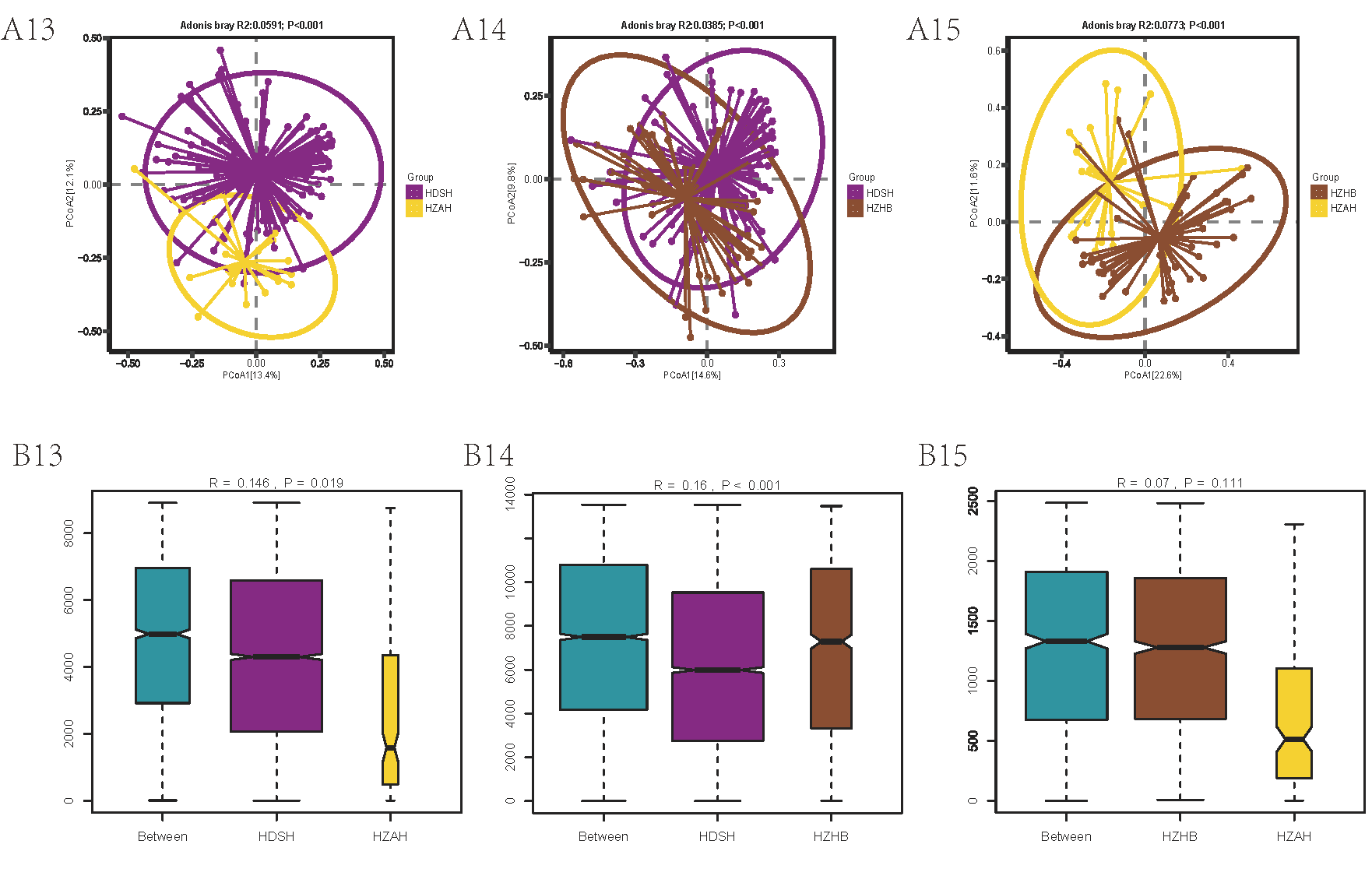


Figure S3. A1-A15, pairwise comparison results between cohorts from different regions based on PCoA (Bray-Curtis); B1-B15, analysis of inter-group and intra-group differences between cohorts from different regions based on ANOSIM test.
